# Supplementary material for: Associations between Phosphate Concentrations and Hospital Mortality in Critically Ill Patients Receiving Mechanical Ventilation
Source: J Clin Med. 2022 Mar 29;11(7):1897. doi: 10.3390/jcm11071897 (PMC8999466; doi:10.3390/jcm11071897)
Supplement: Supplementary file 1 [file jcm-11-01897-s001.zip › jcm-1615706-supplementary.pdf]

Supplementary Table S1. The 2x2 table of hospital mortality predicted by cut-off values calculated by Youden index.

		Death (n=118)	Survival (n=57)	Total (n=175)
Initial	Survival	86 (72.9%)	29 (50.9%)	115 (65.7%)
phosphate*	Death	32 (27.1%)	28 (49.1%)	60 (34.3%)
Delta	Survival	115 (97.5%)	43 (75.4%)	158 (90.3%)
phosphate**	Death	3 (2.5%)	14 (24.6%)	17 (9.7%)
Mean	Survival	104 (88.1%)	24 (42.1%)	128 (73.1%)
phosphate†	Death	14 (11.9%)	33 (57.9%)	47 (26.9%)

Note: The cut-off values of each phosphate were 3.80 mg/dL, 5.58 mg/dL, and 3.70 mg/dL, respectively
* Initial phosphate: the first phosphate value measured within 24 h of admission to the ICU.
** Delta phosphate: the range of change obtained by subtracting the minimum phosphate concentration from the maximum phosphate concentration measured during the ICU stay.
† Mean phosphate: the arithmetical mean value for phosphate concentrations measured during the ICU stay.
